# The evolution of vimentin and desmin in *Pectoralis major* muscles of broiler chickens supports their essential role in muscle regeneration

**DOI:** 10.3389/fphys.2022.970034

**Published:** 2022-09-05

**Authors:** Francesca Soglia, Martina Bordini, Maurizio Mazzoni, Martina Zappaterra, Mattia Di Nunzio, Paolo Clavenzani, Roberta Davoli, Adele Meluzzi, Federico Sirri, Massimiliano Petracci

**Affiliations:** ^1^ Department of Agricultural and Food Sciences (DISTAL), Alma Mater Studiorum–University of Bologna, Bologna, Italy; ^2^ Department of Veterinary Medical Sciences, Alma Mater Studiorum–University of Bologna, Bologna, Italy; ^3^ Department of Food, Environmental and Nutritional Sciences (DeFENS), University of Milan, Milan, Italy

**Keywords:** broiler chicken, growth-rate, immunohistochemistry, western blot, gene expression

## Abstract

Vimentin (VIM) and desmin (DES) are muscle-specific proteins having crucial roles in maintaining the lateral organization and alignment of the sarcomeric structure during myofibrils’ regeneration. The present experiment was designed to ascertain the evolution of VIM and DES in *Pectoralis major* muscles (PM) of fast-growing (FG) and medium-growing (MG) meat-type chickens both at the protein and gene levels. MG broilers were considered as a control group whereas the evolution of VIM and DES over the growth period was evaluated in FG by collecting samples at different developmental stages (7, 14, 21, 28, 35, and 42 days). After performing a preliminary classification of the samples based on their histological features, 5 PM/sampling time/genotype were selected for western blot, immunohistochemistry (IHC), and gene expression analyses. Overall, the findings obtained at the protein level mirrored those related to their encoding genes, although a potential time lag required to observe the consequences of gene expression was evident. The two- and 3-fold higher level of the VIM-based heterodimer observed in FG at d 21 and d 28 in comparison with MG of the same age might be ascribed to the beginning and progressive development of the regenerative processes. This hypothesis is supported by IHC highlighting the presence of fibers to co-expressing VIM and DES. In addition, gene expression analyses suggested that, unlike *VIM* common sequence, *VIM* long isoform may not be directly implicated in muscle regeneration. As for DES content, the fluctuating trends observed for both the native protein and its heterodimer in FG might be ascribed to its importance for maintaining the structural organization of the regenerating fibers. Furthermore, the higher expression level of the *DES* gene in FG in comparison with MG further supported its potential application as a marker of muscle fibers’ regeneration. In conclusion, the findings of the present research seem to support the existence of a relationship between the occurrence of muscle regeneration and the growth rate of meat-type chickens and corroborate the potential use of VIM and DES as molecular markers of these cellular processes.

## Introduction

Vimentin (VIM) and desmin (DES) belong to the family of type III intermediate filament proteins, specific components of the cytoskeletal network having a diameter that is intermediate between those of actin microfilaments and microtubules (Banwell, 2001). These proteins exert a pivotal role in maintaining the lateral organization and alignment of the myofibrils in developing and mature myotubes (Gard and Lazarides, 1980). In detail, although after their synthesis VIM and DES exhibit a diffused cytoplasmic distribution (Vater et al., 1994), upon myofibers’ maturation they attain a sarcomeric pattern and are mainly located at the Z-disk (Bornemann and Schmalbruch, 1992; Vater et al., 1994; Vaittinen et al., 2001). In support of their strong interconnection and essential role in maintaining sarcomeres’ integrity and functionality, VIM and DES were demonstrated to share a common structural organization, comprising a central *α*-helical rod domain–characterized by high homology in their amino acid sequences–flanked by amino- and carboxy-terminal domains (Tokuyasu et al., 1984; Cooper and Hausman, 2000). As for their dynamic expression, it is generally held that VIM can be transiently found in the early stages of myotubes differentiation, whereas DES content gradually increases to become the main intermediate filament protein in mature myofibers (Tokuyasu et al., 1984). In detail, since the newly synthesized DES filaments integrate into the pre-existing VIM ones and partially replace them, VIM and DES were found to co-assemble and co-distribute and, as a consequence, the initial distribution of DES mainly reflects that attained by VIM (Granger and Lazarides, 1979; Tokuyasu et al., 1984; Bornemann and Schmalbruch, 1992).

From the beginning of the XX century, several studies have been carried out to evaluate the intracellular organization and dynamic expression of VIM and DES and improve the knowledge concerning their interactions (Bornemann and Schmalbruch, 1992; Gallanti et al., 1992; Cheng et al., 2016). Later on, these aspects have been widely investigated both in humans affected by neuromuscular and myopathic disorders and in artificially induced animal models (Kottlors and Kirschner, 2010; Fröhlich et al., 2016; Agnetti et al., 2021) to ascertain their potential role in regenerating muscles. These studies allowed to shed light on their crucial role in maintaining muscle cytoarchitecture and recognized them as reliable markers for the regenerative processes taking place within the skeletal muscles (Tokuyasu et al., 1985; Gallanti et al., 1992).

A recent study was performed by our research group to evaluate the distribution and expression of VIM and DES in the *Pectoralis major* muscles (PM) belonging to fast-growing (FG) broilers having normal phenotype, as well as in those exhibiting the macroscopic features ascribable to the white striping, wooden breast, and spaghetti meat abnormalities to ascertain their potential involvement in the time-series of events leading to the progressive development of these conditions (Soglia et al., 2020). Indeed, the histological examinations carried out on PM affected by the abovementioned defects highlighted the occurrence of intense regenerative processes along with profound alterations of the connective tissue composing the perimysial septa (Soglia et al., 2019). Given the above and considering that growth-related abnormalities mainly manifest in broilers selected for growth performance parameters (e.g., growth rate, breast meat yield, etc.), the present experiment was designed to ascertain the evolution of VIM and DES in PM of modern chicken hybrids selected for meat production. In detail, the distribution of VIM and DES and the quantification of their expression, both at the protein and gene level, were evaluated in PM of chickens belonging to FG and medium-growing (MG) genotypes. In this context, MG broilers were considered as a control group in light of the allometric growth of their PM that should not imply the development of intense regenerative processes which are commonly observed in FG (Praud et al., 2021). In detail, to improve the current knowledge concerning the evolution of VIM and DES over the growth period of FG birds and assess the eventual differences which might be ascribed to the animals’ growth rate, samples were collected at different developmental stages (i.e., 7, 14, 21, 28, 35, and 42 days of age).

## Materials and methods

A total of 100 one-day-old male chicks, 70 FG and 30 MG both selected for meat production purposes, were allotted to an environmentally controlled poultry facility. The same commercial corn-wheat-soybean basal diet were provided to both genotypes according to a 3-phase feeding program: starter (0–14 days), grower (15–28 days) and finisher (29-end). Feed and water were administered *ad-libitum*. Stocking density (maximum 33 kg/m^2^), birds’ handling, raising, and processing conditions were defined according to the European legislation in force (European Union, 2007; European Union, 2009; European Union, 2010). The experiment was approved by the Ethical Committee of the Italian Ministry of Health (ID: 1194/2021).

As part of the experimental design, 10 FG and 5 MG birds were slaughtered when reaching different developmental stages (corresponding to 7, 14, 21, 28, 35, and 42 days) and samples were excised from the ventral surface of the PM (facing the skin), following the procedure described by Soglia et al. (2020). Briefly, samples collected for histology and immunohistochemical analyses were excised from the superficial section of the cranial portion of the PM, quickly frozen in isopentane (cooled with liquid nitrogen) and stored at -80°C. Further two samples were collected from the same position of each PM, quickly frozen in liquid nitrogen, and stored in a deep freezer (-80°C) until proteins and RNA extraction.

At each sampling time, PM were preliminary classified according to their macroscopic traits as unaffected muscles, exhibiting macroscopically normal appearance, or affected cases (i.e., showing features ascribable to the white striping and wooden breast abnormalities which are currently affecting the pectoral muscles of the FG hybrids) according to the criteria recently revised by Petracci et al. (2019). Then, before processing the samples for further analyses, a microscopic examination was performed to provide a more accurate and reliable classification of the samples not only based on their macroscopic traits but also considering their histological features (i.e., presence of necrotic fibers and inflammatory cells, increased deposition of connective tissue and fat). From each PM, 10 serial cross-sections (10 μm-thick) were cut on a cryostat microtome at −20°C, mounted on poly-l-lysine coated glass slides (Sigma-Aldrich, St. Louis, MO, United States) and stained with hematoxylin and eosin. Then, after evaluating their histological features, 5 PM/sampling time/genotype were selected and considered for further analyses.

### Immunohistochemistry

Immunohistochemical analyses were performed following the procedure described in our previous study (Soglia et al., 2020) based on the avidin-biotin-peroxidase complex (ABC) method, with slight modifications. Briefly, for each PM, 10 serial cross-sections (10 μm thick) were cut on a cryostat microtome and mounted on poly-l-lysine coated glass slides (Sigma–Aldrich, St. Louis, MO). After rinsing in phosphate buffer saline, sections were incubated in 5% normal goat serum (for 30 min at room temperature, RT) to limit the eventual non-specific binding of the secondary antibodies. Then, sections were incubated (4°C in humid chamber for 24 h) with a monoclonal mouse anti-VIM and a polyclonal rabbit anti-DES (61013 and 10570, Progen Biotechnik GmbH, Heidelberg, Germany, respectively) antibodies both diluted 1:1000. After washing, sections were incubated (RT for 1 h) with the biotin-conjugated goat anti-mouse IgG and biotin-conjugated goat anti-rabbit IgG secondary antibodies, both diluted 1:200 (Vector Laboratories, Burlingame, CA, USA), and then treated with ABC (Vector elite kit, Vector Laboratories, Burlingame, CA, USA). The immune reactions were then visualized through the 3,3′-diaminobenzidine (DAB) chromogen solution supplied by Vector (Vector DAB kit, Vector Laboratories, Burlingame, CA, USA). The sections were then washed in PBS and coverslipped with buffered glycerol, pH 8.6.

### Western blot

Myofibrillar proteins were extracted following the procedure described by (Liu et al., 2014) with slight modifications. Briefly, 1 G of frozen PM was homogenized by Ultra-Turrax (IKA, Germany) (20 s at 13,500 rpm, in ice) in 20 ml of cold Rigor Buffer (75 mM KCl, 10 mM KH_2_PO_4_, 2 mM MgCl_2_, 2 mM EGTA; pH 7.0) (Sigma–Aldrich, St. Louis, MO). The homogenate was centrifuged (10 min at 10,000 × g at 4°C) and the supernatant, containing the sarcoplasmic protein fraction, was discarded. The same procedure was repeated by adding 10 ml of cold Rigor Buffer, homogenizing (20 s at 13,500 rpm, in ice) and centrifuging the samples for 20 min under the same conditions previously adopted (10,000 × g at 4°C) and the resultant pellet, containing the myofibrillar proteins, was re-suspended by homogenization in 10 ml of cold Rigor Buffer. After being quantified (Bradford, 1976) by using bovine serum albumin as standard, the protein content of each extract was adjusted to 2.0 mg/ml and each sample was mixed 1:1 (v/v) with Sample Buffer (50 mM Tris-HCl, 8 M Urea, 2 M Thiourea, 75 mM DTT, 3% (v/v) SDS; pH 6.8) (Sigma–Aldrich, St. Louis, MO) (Fritz et al., 1989).

Myofibrillar proteins (10 μg) were loaded in 4–15% Mini-PROTEAN TGX Stain-Fee™ Gels (Bio-Rad Laboratories) and the electrophoretic separation was carried out with a Bio-Rad Mini Protean II electrophoresis apparatus at constant voltage (200 V) for 30 min. Gels were subsequently activated by UV exposure to produce a fluorescent signal resulting from the interaction of the tryptophan residues in the proteins polypeptide chains with the trihalo compounds incorporated into gel formulation (Gürtler et al., 2013), and protein fluorescence was acquired (to check the electrophoretic separation of the proteins) using a ChemiDoc™ MP Imaging System (Bio-Rad Laboratories) with the Image Lab software (version 5.2.1). Proteins were transferred onto a 0.2 µm nitrocellulose membrane using a Trans-Blot^®^ Turbo^™^ Transfer System (Bio-Rad Laboratories) and incubated (45 min, at room temperature while shaking) with 15 ml TBST (Tris Buffered Saline with Tween^®^ 20–20 mM Tris, 150 mM NaCl, 0.1% Tween 20; pH 7.4–7.6) with 5% skimmed milk powder. Membranes were probed (60 min, room temperature while shaking) with a monoclonal mouse anti-VIM (61013, Progen Biotechnik GmbH, Heidelberg, Germany) and a polyclonal rabbit anti-DES (10570, Progen Biotechnik GmbH, Heidelberg, Germany) antibodies diluted 1:4,000 and 1:6,000, respectively. After washing, the membranes were incubated with secondary anti-mouse and anti-rabbit antibodies for 60 min (1:15,000) (Merk Millipore, Burlington, Massachusetts, USA) and treated with Horseradish Peroxidase (HRP)-conjugated streptavidin (Merk Millipore, Burlington, Massachusetts, USA) for 20 min. Final detection was performed with enhanced chemiluminescence (Clarity™ Western ECL Substrate) Western Blotting detection kit (Bio-Rad Laboratories) and the images were acquired using the ChemiDoc™ MP Imaging System (Bio-Rad Laboratories). Densitometry differences were analyzed with the Image Lab software and normalized for the total fluorescent protein signal intensity (Valli et al., 2018). The results were expressed as %, considering 100% the intensity of the band assigned to VIM and DES in the PM belonging to the MG genotype sampled on d 7.

### Gene expression

Total RNA was extracted from frozen PM muscles using TRIzol^®^ Reagent (Invitrogen™, Thermo Fisher Scientific, Waltham, MA, United States), following the manufacturer’s instructions. Quantification and purity of extracted RNA were tested by a ND-1000 Spectrophotometer (NanoDrop Technologies, Wilmington, DE, United States). Also, a visualization by agarose 1% was performed to check RNA integrity (Zappaterra et al., 2015). Then, iScript™ gDNA Clear cDNA Synthesis Kit (1725035, Bio-Rad Laboratories) was used for removing genomic DNA (gDNA) contamination from each sample and retrotranscribing 1 μg of total RNA to complementary DNA (cDNA), as recommended by the manufacturers. The expression levels of *Desmin* (*DES*) and two *Vimentin* sequences (*Vimentin* long isoform - *VIM long; Vimentin* common sequence - *VIM com*), as described in our previous study (Soglia et al., 2020), have been analyzed in the present paper. In particular, target and normalizing genes quantifications have been assessed by relative quantitative Real-Time polymerase chain reaction (qRT-PCR), using the standard curve method (Pflaff et al., 2004) on Rotor-Gene™ 6,000 (Corbett Life Science, Concorde, NSW, Australia). The qRT-PCRs were performed in a total volume of 10 μl using SsoAdvanced™ universal SYBR^®^ Green Supermix (1725271, Bio-Rad Laboratories). Primer pairs were designed using Primer3Plus web online tool (Untergasser et al., 2012) and their complete information is reported in Supplementary Table S1. For each sample, every gene was quantified in triplicate to collect sample replications with coefficients of variation lower than 0.2. As regards the data normalization, genes coding for the ribosomal protein L4 (*RPL4*), ribosomal protein lateral stalk subunit P0 (*RPLP0*) and glyceraldehyde-3-phosphate dehydrogenase (*GAPDH*), which are reported as housekeeping in several studies (Velleman et al., 2014; Bagés et al., 2015; Powell et al., 2016; Zhang et al., 2018) were tested to identify the best couple of normalizing genes. In our study, *RPL4* and *GAPDH* were used as reference genes for the normalization of relative quantification of target genes, since the GeNorm algorithm (Vandesompele et al., 2002) evidenced them as the most stable genes. Amplification conditions of target and normalizing genes are reported in Supplementary Table S2.

### Statistical analysis

Data were analyzed by using Statistica 10 (StatSoft Inc., 2014). In detail, within each sampling age (i.e., 7, 14, 21, 28, 35, and 42 days), the non-parametric Mann-Whitney U test was applied to assess the effect of the genotype (FG vs. MG) on the findings achieved for VIM and DES both at protein and gene level. For FG broilers, the one-way ANOVA option was used to evaluate the evolution of VIM and DES proteins as well as of their coding genes over their growth period. In addition, Spearman’s correlations among the gene expression of *DES* and the two *VIM* sequences (*VIM long* and *VIM com*) were calculated per each sampling age and considering FG broilers on their whole. All statistical differences were considered significant at a level of *p* ≤ 0.05.

## Results

### Histology

**FIGURE 1:**
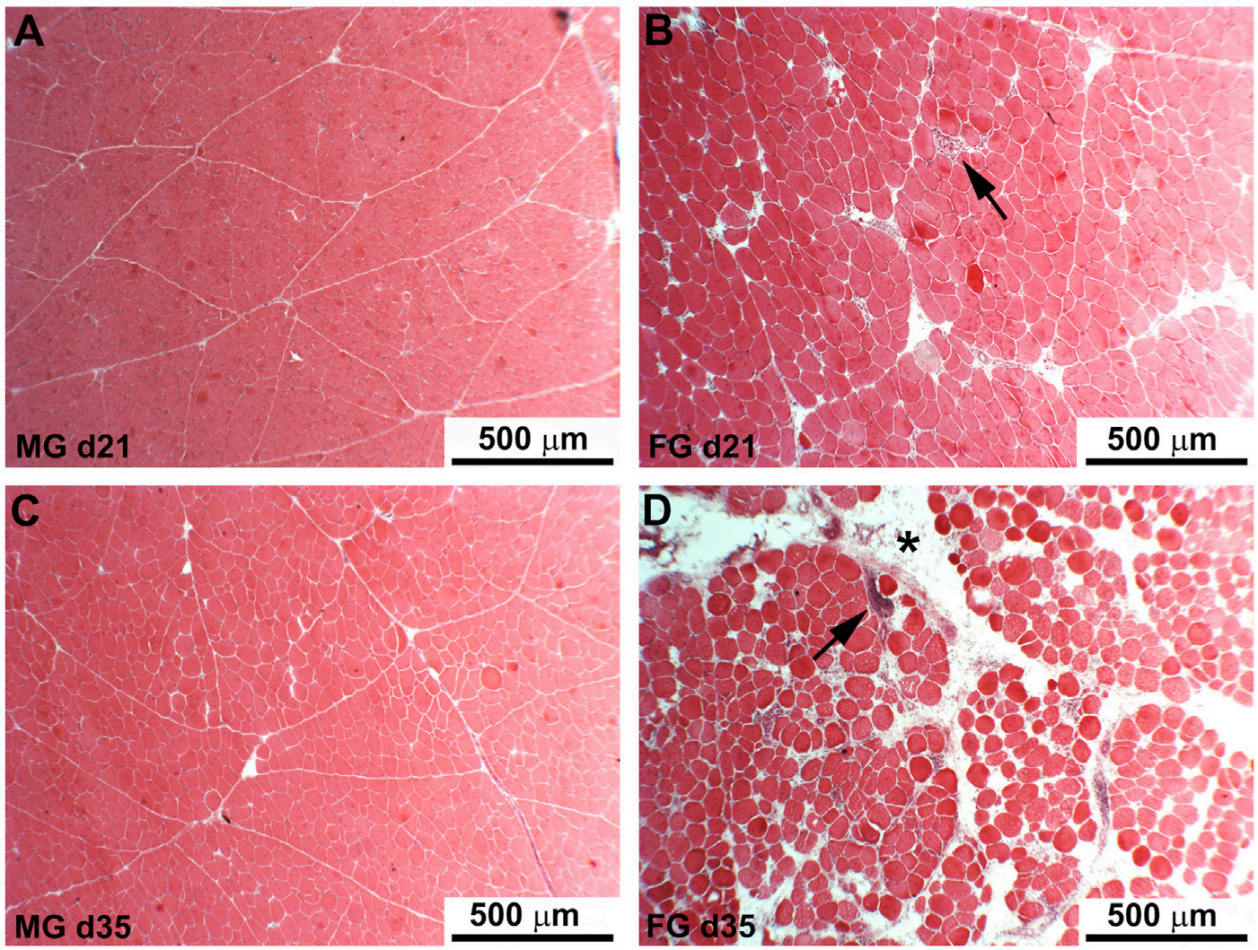
Hematoxylin and eosin staining of the *Pectoralis major* muscles of medium-growing chickens (21 and 35 days old, respectively) show fibers having a regular polygonal profile surrounded by normal endomysium and perimysium without any detectable damage or additional deposit of intermuscular adipose tissue **(A,C)**. In fast-growing chickens **(B,D)** many of the fibers exhibited rounded profile and variable diameter along with the presence of abundant adipose tissue infiltrated at endomysial and perimysial (D, asterisk) levels. Arrows indicate necrotic fibers surrounded by inflammatory infiltrates.

The results of the histological observations performed on PM of FG and MG chickens at different developmental stages (i.e., 7, 14, 21, 28, 35, and 42 days) are shown in Figure 1. A normal muscular architecture (Figure 1A,C), including fibers exhibiting polygonal profile and a normally structured connective tissue composing the endomysial and perimysial septa, was observed in MG during their whole growth period. As for FG, the histological features observed in the early stages of muscle development (i.e., 7, 14 and 21 days of age) were similar to those observed in MG whereas a prevalence of fibers having rounded profile along with a profoundly altered endomysial and perimysial connective tissue were found as the developmental stages proceeded (at d 28, 35, and 42). At these ages, several muscle fibers exhibited nuclear internalization, variable cross-sectional area (suggesting the contextual presence of degenerating and regenerating fibers), necrosis up to lysis, inflammatory cell infiltration, lipidosis, and fibrosis (Figures 1B,D).

### Immunohistochemistry

**FIGURE 2:**
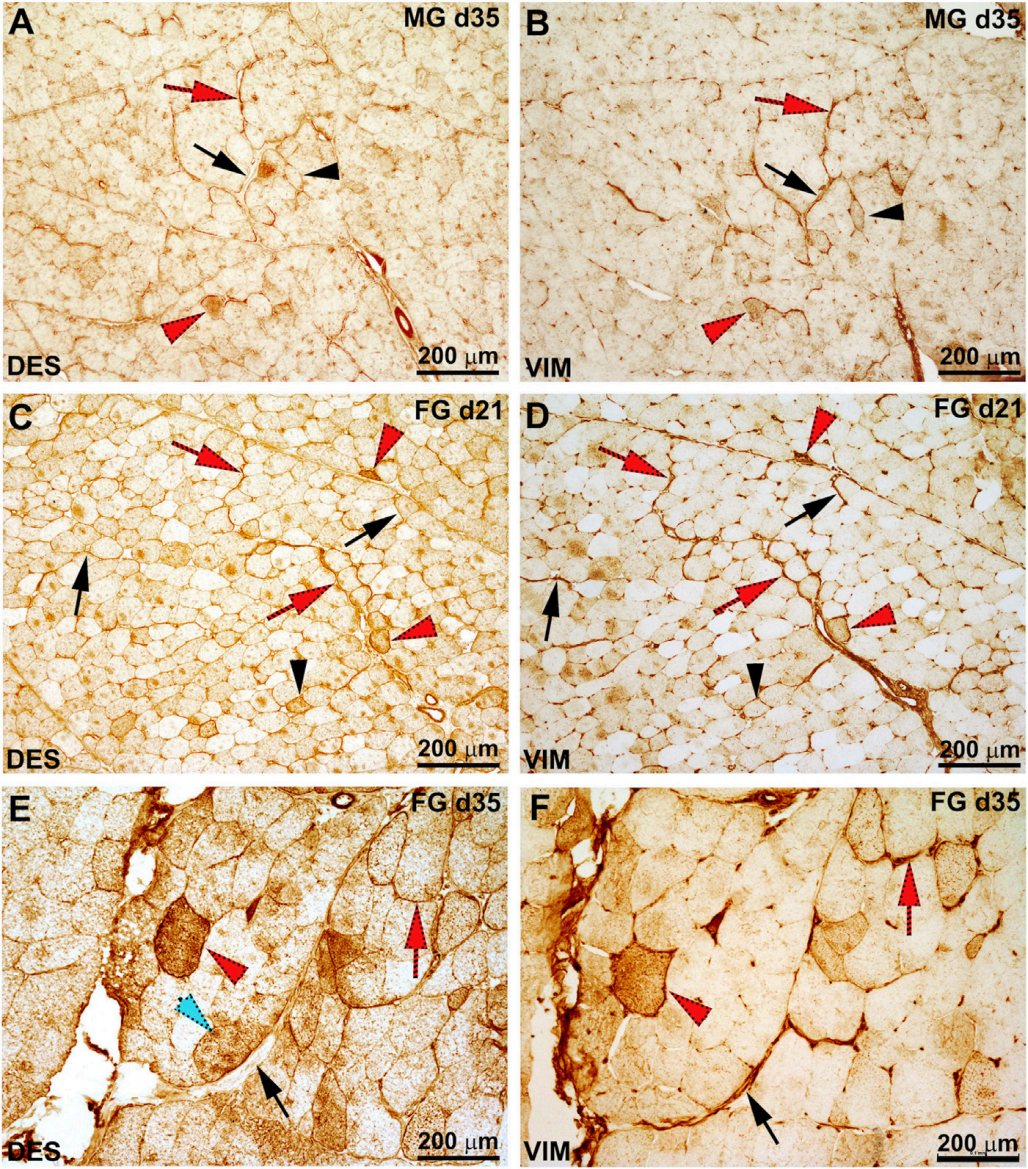
Representative images of the serial sections for desmin (DES) and vimentin (VIM) immunoreactivity in the medium-growing (MG) **(A, B)** and fast-growing (FG) **(C–F)** chickens at 21 and 35 days of age. The perimysial connective showed positivity against each target protein, while in other areas of the sections immunoreactivity against one protein rather that the other was evident. In detail, some tracts of the perimysial connective were found to co-express DES and VIM (red arrow) while in others only immunoreactivity for VIM was observed (black arrow). Similarly, some fibers were found to colocalize DES and VIM (red arrowhead) while others exhibit positivity only for VIM or DES (black arrowhead).

Representative images of the pattern of immunoreactivity for VIM and DES in FG and MG chickens are shown in Figure 2. Overall, reactivity against VIM and DES was mainly observed in the intermyofibrillar network (peri and endomysial connective tissue), blood vessels and, in some cases, at the level of the fibers themselves. In detail, aside from the genotype, immunoreaction within the connective tissue was found particularly intense between 21 and 35 days of age. In addition, segments of the connective tissue composing the endo and perimysial septa as well as some fibers were found to co-express VIM and DES, whereas others exhibited a distinct reaction for VIM or DES (Figure 2A–F). As for their localization, VIM and DES were in some cases confined in the sub-sarcolemmal position while in others immunoreactivity was homogeneously distributed throughout the sarcoplasm (Figure 2). Regarding the effect of animals’ growth, the greatest number of fibers immunoreactive to VIM and/or DES was observed at d 21, 28 and 35 (aside from the genotype), while a lower number of positive fibers was found earlier (i.e., d 7 and d 14) and at the end of the farming period (i.e., d 42).

### Western blot

**FIGURE 3:**
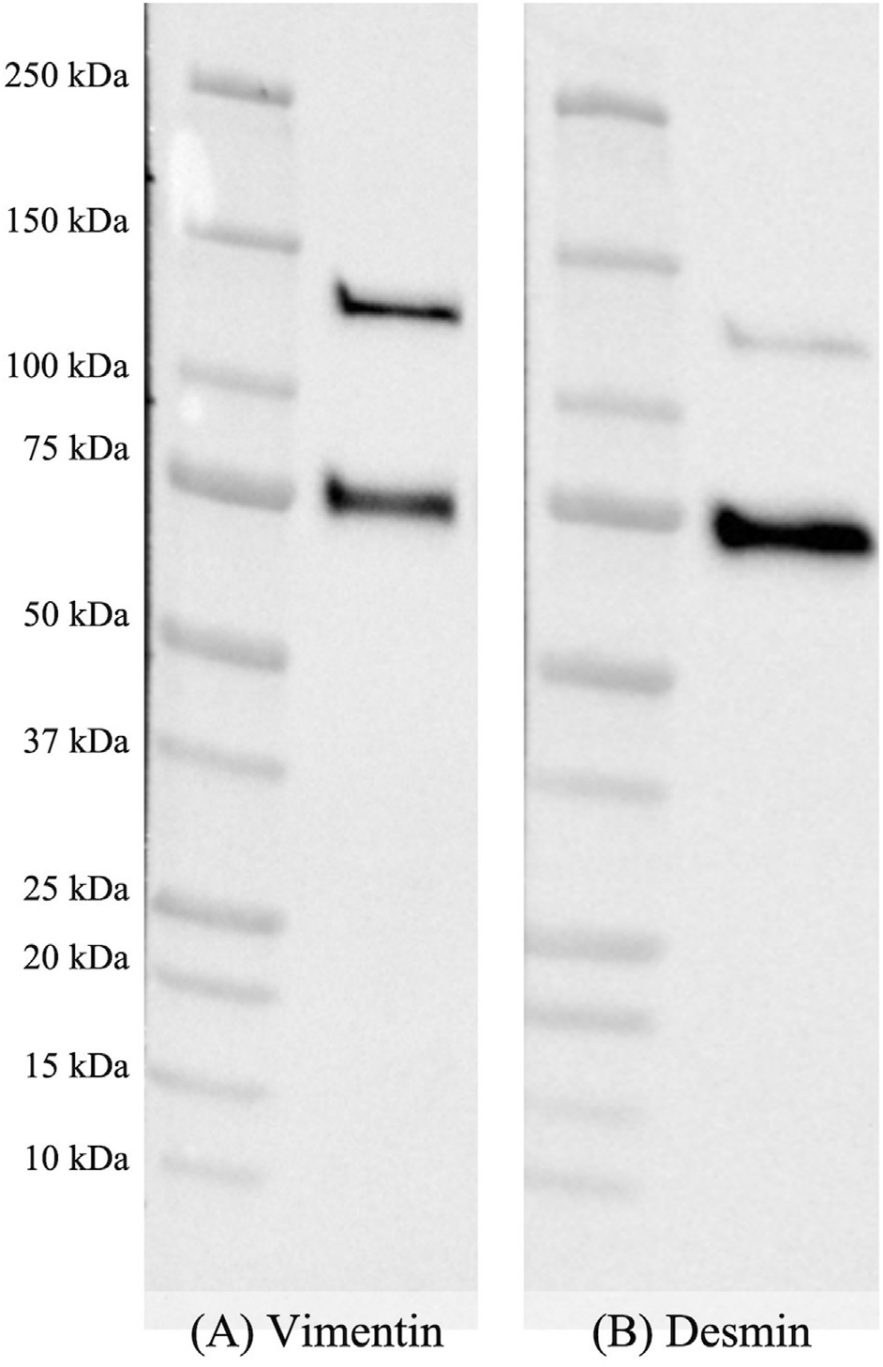
Representative image of nitrocellulose membranes incubated with monoclonal mouse anti-vimentin **(A)** and polyclonal rabbit anti-desmin **(B)** primary antibodies after performing the final detection with enhanced chemiluminescence.

A representative image of the immunoblots obtained for VIM and DES is shown in Figure 3A,B (See also Supplementary Figure S3). Two distinct bands having, according to the marker, a molecular weight of 65 and 130 kDa were identified and ascribed to the native proteins (i.e., VIM and DES) and to their heterodimeric forms (i.e., VIM-VIM, DES-DES, VIM-DES).

**FIGURE 4:**
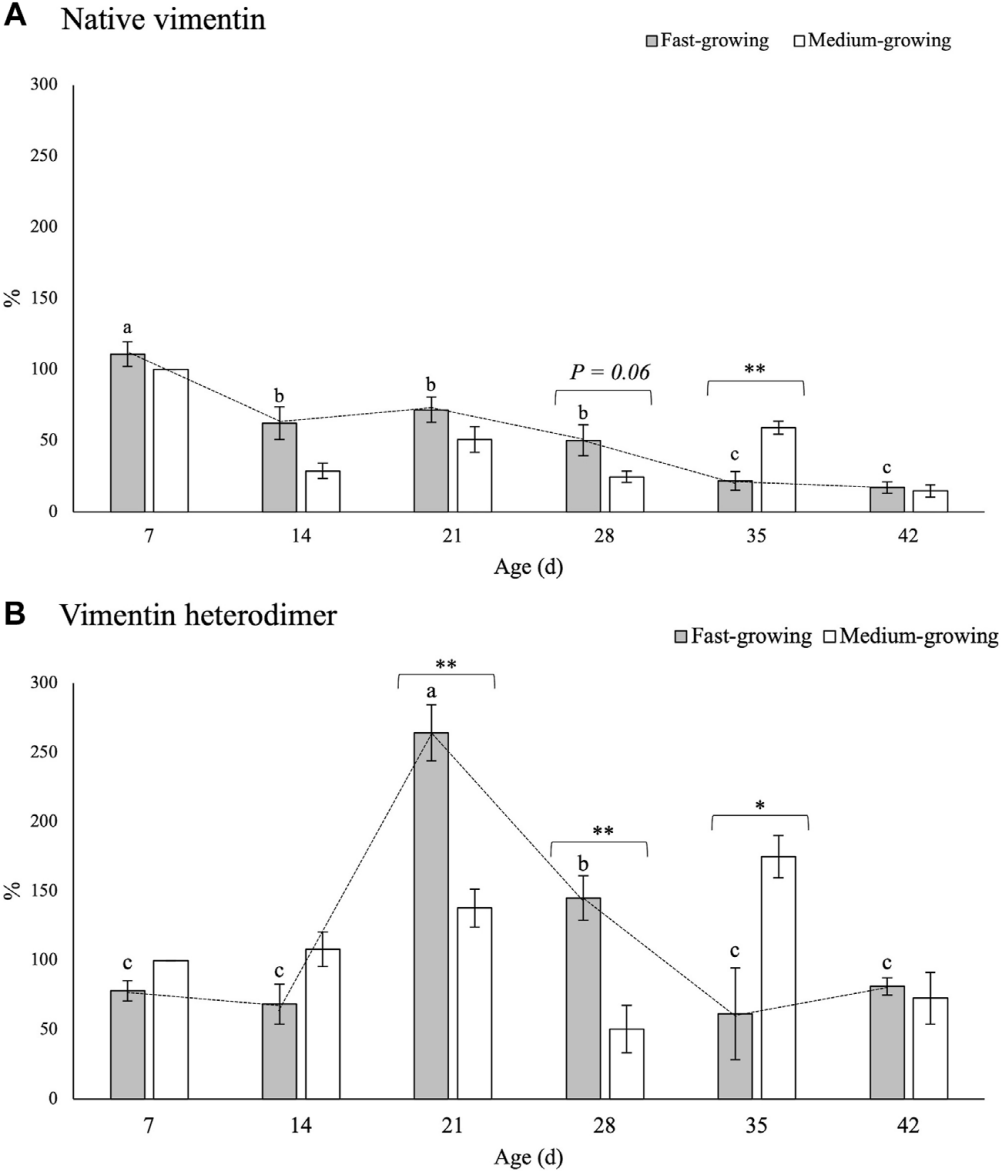
Relative concentrations of native vimentin **(A)** and of its heterodimeric form **(B)** in *Pectoralis major* muscles of broiler chickens belonging to fast- and medium-growing genotypes at different ages. The results are expressed as %, considering as 100% the intensity of the band in MG at the beginning of the rearing period (day 7–d 7). Error bars indicate standard error of mean. Within the same age (7, 14, 21, 28, 35, and 42 days) data were analyzed by using the non-parametric Mann-Whitney U test to investigate the effect of the genotype (FG vs. MG). * and ** = within the same age, mean values significantly differ between FG and MG for a *p*-value <0.05 and <0.01, respectively. On the other hand, the evolution of vimentin in FG over their growth period was assessed by One-way ANOVA. a-c = for FG, mean values followed by different letters significantly differ over the growth period (*p* < 0.05).

**FIGURE 5:**
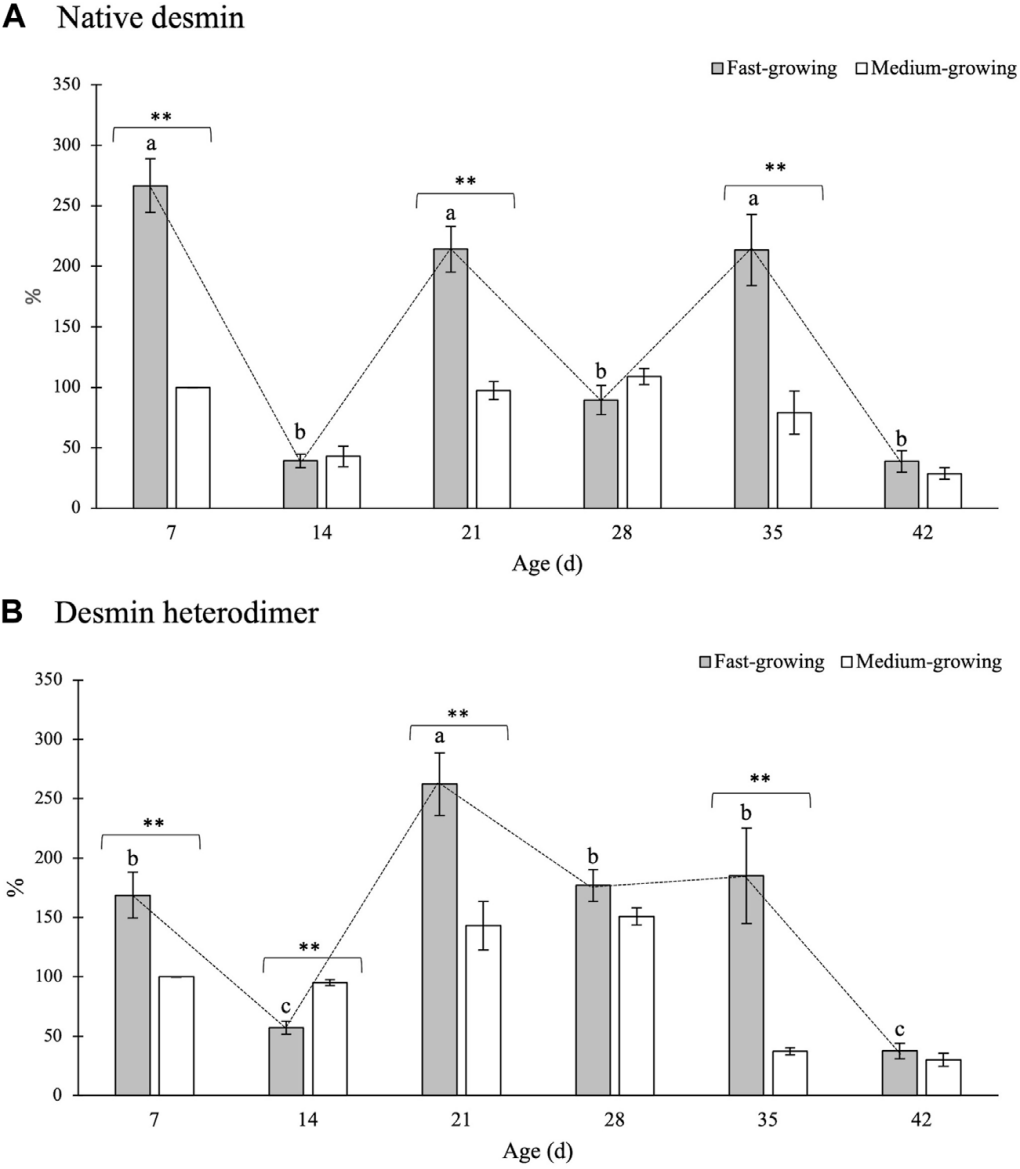
Relative concentrations of native desmin **(A)** and of its heterodimeric form **(B)** in *Pectoralis major* muscles of broiler chickens belonging to a fast- and medium-growing genotype at different ages. The results are expressed as %, considering as 100% the intensity of the band in MG at the beginning of the rearing period (day 7–d 7). Error bars indicate standard error of mean. Within the same age (7, 14, 21, 28, 35, and 42 days) data were analyzed by using the non-parametric Mann-Whitney U test to investigate the effect of the genotype (FG vs. MG). * and ** = within the same age, mean values significantly differ between FG and MG for a *p*-value <0.05 and <0.01, respectively. On the other hand, the evolution of desmin in FG over their growth period was assessed by One-way ANOVA. a-c = for FG, mean values followed by different letters significantly differ over the growth period (*p* < 0.05).

The findings concerning the expression levels of VIM and DES in PM of FG and MG broilers at different developmental stages (7, 14, 21, 28, 35, and 42 days of age) are reported in Figures 4A,B and Figures 5A,B, respectively.

No significant differences among the genotypes were found in the relative concentration of the 65-kDa band with the only exception being the expression level of native VIM assessed at d 28 and d 35 of age (Figure 4A). Indeed, if compared with the results obtained in MG at d 28, a tendency (*p* = 0.06) of a higher VIM content was found in FG (25 vs. 50%) whereas its expression level was reduced to less than one half at d 35 (59 vs. 22%; *p* < 0.01).

As for the effect of the developmental stage, a progressive decline (*p* < 0.001) in the relative concentration of native VIM was observed within the PM of FG chickens as the age increased. In detail, if compared with the findings obtained at d 7, a significant reduction (-85%) in the relative concentration of native VIM was observed at d 42 (111 vs. 17%; *p* < 0.001).

The findings concerning the relative concentration of the VIM heterodimeric form are shown in Figure 4B. No significant differences between FG and MG were found either at the beginning (i.e., d 7 and d 14) or at the end (d 42) of the rearing period. On the other hand, if compared with MG, two- and 3-fold higher (*p* < 0.01) expression levels of the 130-kDa band were found in FG at d 21 and 28, respectively. On the other hand, in agreement with the findings obtained for the native protein, a significantly higher (*p* < 0.05) relative concentration of the heterodimeric form was found at d 35 in MG in comparison with FG.

As for the effect ascribable to the developmental stage, the expression level of the 130-kDa band was found to sharply increase from d 14 to d 21, in which the highest values were found, followed by a progressive decline in the following ages.

The outcomes concerning the relative concentration of native DES and its heterodimeric form are reported in Figure 5A,B, respectively.

Native DES was found to be significantly higher within the PM belonging to FG if compared with MG at 7, 21, and 35 days of age (Figure 5A). On the other hand, no significant differences ascribable to the genotype were observed for the other developmental stages considered in the present study (i.e., 14, 28, 42 days).

As for the evolution of the content of native DES at different developmental stages of the birds, a wavering trend was found: a remarkable increase in native DES observed at 7, 21, and 35 days was followed by a sharp decline in its content in the following ages (i.e., 14, 28, and 42 days). A similar trend was observed for the 130-kDa band (Figure 5B). In detail, if compared with MG, a remarkably higher content of the heterodimeric form was measured in FG at 7, 21, and 35 days (100 vs. 169%, 143 vs. 262%, and 37 vs. 185%; *p* < 0.01, respectively). No significant differences ascribable to the genotype were found at d 28 and d 42 whereas a significantly higher content of the heterodimer was observed at d 14 in MG in comparison with FG broilers of the same age (95 vs. 57%; *p* < 0.01).

As for the effect of the developmental stage, a significant decline in the relative concentration of the heterodimer was found at d 14 followed by a sharp increase in the concentration of this electrophoretic fragment (d 21). Then, the content of the 130-kDa band progressively declined as the age increased with the values assessed at the end of the rearing period being 4-fold lower than those measured at the beginning of the trial.

### Gene expression

**FIGURE 6:**
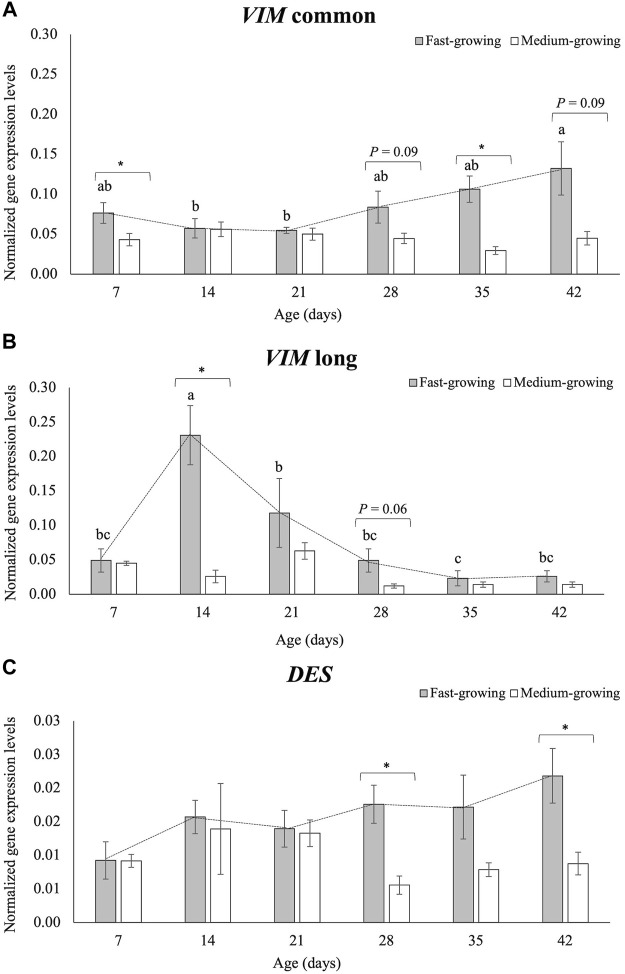
The normalized expression values of the *Vimentin* (*VIM*) common sequence **(A)**, *Vimentin* (*VIM*) long-isoform **(B)**, and *Desmin* (*DES*) gene **(C)** in *Pectoralis major* muscles of broilers belonging to a fast- and medium-growing genotype at different ages (7, 14, 21, 28, 35, and 42 days). For each panel **(A,B,C)**, the bar graphs show the mean values of the normalized expression level for each genotype (FG and MG) at each sampling time, and the error bars indicate the measured standard deviations. Significant differences between FG and MG at each sampling time were analyzed by using the non-parametric Mann-Whitney U. The significant *p*-values (*p* ≤ 0.05; *) and trends towards significance (*p* ≤ 0.10) are reported for the comparisons between FG and MG within each sampling age. On the other hand, the line graph evidences the evolution of the normalized expression level of each gene during the time in FG broilers. The evolution of the normalized gene expression of *VIM* common sequence, *VIM* long-isoform and *DES* gene in FG over their growth period was assessed by One-way ANOVA. a-c = for FG, mean values followed by different letters significantly differ over the growth period (*p* ≤ 0.05).

The normalized quantifications of the two *VIM* sequences (i.e., *VIM com* and *VIM long*) and the *DES* gene at each sampling time (7, 14, 21, 28, 35, and 42 days of age) are reported in Figure 6.

The *VIM* common sequence showed a normalized expression level higher in FG than in MG broilers at d 7 (*p* < 0.05) and d 35 (*p* < 0.05), and the difference was close to being significant at d 28 and d 42 (*p* = 0.09). No differences in the *VIM com* gene expression between FG and MG were found at d 14 and d 21 (Figure 6A). As for the *VIM long* (Figure 6B), the normalized expression level obtained using the primers specific for the *VIM* long-isoform showed significant differences between FG and MG at d 14 (*p* < 0.05), while a tendency was found at d 28 (*p* = 0.06). The normalized quantification of the *DES* gene is reported in Figure 6C *. DES* mRNA level was significantly higher expressed in FG than in MG broilers at d 28 (*p* < 0.05) and 42 (*p* < 0.05), whereas no differences were observed at d 7, d 14, d 21 and d 35.

As for the evolution of the normalized expression of the two *VIM* sequences in FG during the growth period (Table 1 and Figure 6A,B), transcription levels of *VIM com* progressively increased when passing from d 14 and d 21 to d 42. On the contrary, *VIM long* showed a progressive decline from d 14 to d 42. As for the effect of the developmental stage on the *DES* normalized expression in FG broilers, no statistical differences have been found (Table 1 and Figure 6C).

**TABLE 1:** One-way ANOVA results for the different developmental stages of fast-growing (FG) broilers. For each sampling time (d 7, d 14, d 21, d 28, d 35 and d 42), mean and standard deviation (SD) of the normalized gene expressions are reported.

FG					
Gene	N	Mean	SD	Group effect	
				F Value	*P* (>F)
***VIM long***				6.792	0.001
d 7	5	0.049	0.017		
d 14	5	0.231	0.043		
d 21	5	0.118	0.050		
d 28	5	0.049	0.017		
d 35	5	0.023	0.011		
d 42	5	0.026	0.008		
***VIM com***				2.386	0.069
d 7	5	0.076	0.013		
d 14	5	0.057	0.012		
d 21	5	0.055	0.004		
d 28	5	0.084	0.020		
d 35	5	0.106	0.016		
d 42	5	0.132	0.033		
***DES***				1.541	0.214
d 7	5	0.009	0.003		
d 14	5	0.016	0.002		
d 21	5	0.014	0.003		
d 28	5	0.018	0.003		
d 35	5	0.017	0.005		
d 42	5	0.022	0.004		

All the Spearman’s correlation results obtained considering the normalized expression level of *VIM com*, *VIM long* and *DES* in FG broilers are reported in Table 2. Considering all the sampling times together, the normalized transcription levels of the two *VIM* sequences in FG broilers resulted to be negatively related to each other (r = -0.58; *p* < 0.01). Furthermore, Spearman’s correlation analysis performed considering the sampling time separately showed a positive correlation between *VIM long* and *DES* in PM belonging to FG broilers at d 35 (r = 0.90; *p* < 0.05). On the other hand, the normalized quantification of *DES* in FG broilers resulted positively correlated to *VIM com* at d 42 (r = 0.90; *p* < 0.05).

**TABLE 2:** Spearman’s correlations between the expression levels of *VIM* common sequence (*VIM com*), *VIM* long-isoform (*VIM long*) and *DES* genes in FG broilers considering all the samples together and each sampling time separately (d 7, d 14, d 21, d 28, d 35 and d 42). Significant correlations are reported in bold. *p < 0.05; **p < 0.01; n.s., not significant.

All	*VIM com*	*VIM long*	*DES*
*VIM com*	1	-0.58**	n.s
*VIM long*	-0.58**	1	n.s
*DES*	n.s	n.s	1
**d 7**	*VIM com*	*VIM long*	*DES*
*VIM com*	1	n.s	n.s
*VIM long*	n.s	1	n.s
*DES*	n.s	n.s	1
**d 14**	*VIM com*	*VIM long*	*DES*
*VIM com*	1	n.s	n.s
*VIM long*	n.s	1	n.s
*DES*	n.s	n.s	1
**d 21**	*VIM com*	*VIM long*	*DES*
*VIM com*	1	n.s	n.s
*VIM long*	n.s	1	n.s
*DES*	n.s	n.s	1
**d 28**	*VIM com*	*VIM long*	*DES*
*VIM com*	1	n.s	n.s
*VIM long*	n.s	1	n.s
*DES*	n.s	n.s	1
**d 35**	*VIM com*	*VIM long*	*DES*
*VIM com*	1	n.s	n.s
*VIM long*	n.s	1	0.90*
*DES*	n.s	0.90*	1
**d 42**	*VIM com*	*VIM long*	*DES*
*VIM com*	1	n.s	0.90*
*VIM long*	n.s	1	n.s
*DES*	0.90*	n.s	1

## Discussion

In the present study, the evolution of VIM and DES in the PM of FG and MG chickens has been assessed to improve the current knowledge concerning their evolution over the growth period of the birds and ascertain their eventual implication in the regenerative processes characterizing the pectoral muscles affected by growth-related abnormalities. The outcomes of the histological evaluations allowed us to obtain an accurate and precise classification of the PM. As expected, the PM of MG exhibited a normal muscular architecture in all the developmental stages considered in the present study whereas, although unaffected up to d 21, the FG ones displayed the microscopic lesions ascribable to white striping and/or wooden breast defects from d 28 onwards. These findings corroborated the evidence of an early onset of growth-related abnormalities in FG broilers, thus confirming that even those PM having a normal phenotype may exhibit microscopic features ascribable to muscular abnormalities (Sihvo et al., 2018; Mazzoni et al., 2020). In addition, the histological examinations seemed to support a strong association between the altered muscular architecture observed in FG broilers during growth and the breeding selection processes/plans which were implemented for their development.

As for the findings achieved through Western Blot analyses, the presence of two bands, having a molecular weight of 65 and 130 kDa, might be ascribed to native VIM or DES and to the development of either homo- or hetero-dimers (i.e., VIM-VIM, DES-DES, VIM-DES) resulting from their association at the Z-disk (Cooper and Hausman, 2000). This hypothesis is corroborated by previous studies demonstrating the existence of a VIM-DES heterodimer, consisting of face-to-face pairs of two different VIM and DES subunits, both in skeletal and smooth muscles as well as in cultured renal cells (Quinlan and Franke, 1982; Traub et al., 1993; Yang and Makita, 1996). Interestingly and consistently with what has been found by Traub et al. (1993), these two IF proteins seem to resist dissociation also using a high concentration of urea (e.g., 8 M urea) in the extraction buffer, thus suggesting a strong protein dimerization.

When considering the findings obtained for VIM protein, the differences related to the genotype might be presumably attributed to the higher growth rate of FG broilers. The two- and 3-fold higher level of the VIM-based heterodimer observed in FG at d 21 and d 28 in comparison with MG chickens of the same age might be ascribed to the beginning and progressive development of the regenerative processes taking place in these PM. Indeed, a sharp increase in VIM synthesis was already observed during the early stages of regeneration (Vater et al., 1994). Although at d 21 none of the PM belonging to FG exhibited the phenotypes and the macroscopic features associated with the white striping and/or wooden breast conditions, that did not exclude the eventual occurrence of occasional, up to intense, regenerative phenomena involving an augmented synthesis of VIM. Indeed, evident signs of muscle regeneration were demonstrated even within those PM exhibiting a normal visual appearance (Mazzoni et al., 2020). The histological observations further corroborated the results obtained by Western Blot analysis. Indeed, the first evidence of abnormalities affecting the fibers and the connective tissue were readily detected in FG at d 21. In agreement with that, the immunohistochemical analyses performed at this age revealed an increased expression of VIM both at the level of the endo and perimysial connective tissue as well as located within the muscle fibers in a sub-sarcolemmal and sarcoplasmic positions. Overall, these findings might support the hypothesis of the occurrence of intense regenerative processes in an early stage within these PM which is in agreement with previous studies carried out on wooden breast-affected muscles (Papah et al., 2017; Sihvo et al., 2017; Griffin et al., 2018; Chen et al., 2019). As for the findings achieved at d 28, if compared with MG, the significantly higher VIM content (either in its native or heterodimeric forms) observed in FG broilers - which from this age exhibited the distinctive features associated with growth-related myopathies - might be due to the progression of the regenerative processes.

As for the effect of the FG birds’ growth, overall, different trends were observed for the native protein and its heterodimeric form. In detail, the progressive reduction in native VIM observed as the developmental stage proceeded might be explained by considering previous studies, carried out at the end of the 1970s, demonstrating a higher VIM content in skeletal muscles belonging to young rather than old chickens (Granger and Lazarides, 1979; Gard and Lazarides, 1980). On the other hand, VIM heterodimeric form exhibited a bell-shaped evolution in which a sharp increase from d 7 and d 14 to d 21 (when it reaches its maximum expression) was then followed by a gradual decline thereafter. These outcomes perfectly matched with those obtained through IHC in which a higher and more intense immunoreactivity for VIM was observed in the inter-myofibrillar network at d 21 and d 35. This trend might be due to the progression of the regenerative processes within the PM requiring the synthesis of this protein. Indeed, VIM is transiently expressed in immature myotubes and, although it gradually disappears upon myoblasts fusion (Bennett et al., 1979; Granger and Lazarides, 1979; Bornemann and Schmalbruch, 1992; Gallanti et al., 1992), it can be detected in fibroblasts composing the connective tissue sheaths as well as in vascular endothelia (Čížková et al., 2009). In agreement with these observations, a 55% higher VIM content was found in wooden breast-affected PM if compared with their unaffected counterpart (Soglia et al., 2020). In this context, it is worth mentioning that VIM seems to be implicated in several cellular processes occurring during muscle regeneration and that the available knowledge concerning their related pathway may help in understanding the mechanisms associated to muscle regeneration in FG broilers. The presence of degenerating/necrotic fibers, indeed, induces the activation of a satellite cell (SC)-mediated regeneration process whose first evidence at molecular and histological levels was observed in FG as early as two- and 3 weeks post-hatch, respectively (Papah et al., 2017, 2018; Sihvo et al., 2017). Intriguingly, this time course seems to perfectly match the findings achieved in the present study and support the hypothesis of a relevant role of VIM in muscle regeneration (Ostrowska-Podhorodecka et al., 2022) which, in its turn, contributes to explain the absence of differences at the protein level between FG and MG birds at the beginning of the growing period. This initial similarity may be partially due to the response time needed to induce the development of the first regenerative processes to counteract myofiber degeneration and necrosis. Indeed, VIM participates in many processes of crucial importance for tissue repair and regeneration (Cheng et al., 2016; Danielsson et al., 2018) and its expression is maximal during myotubes formation and SCs proliferation (Paulin et al., 2022). In this regard, the findings of the present study demonstrated that the occurrence of regenerative processes within the PM of FG birds can be detected at protein level (either by means of Western Blot or IHC analyses) as early as 3 weeks post-hatch when the content of the VIM-based heterodimer reaches its utmost level.

When considered on their whole, findings at protein level mirrored those related to gene expression. In detail, any change observed in the expression level of the *VIM* and *DES* genes seemed to be followed by a variation in protein synthesis in the subsequent sampling time, thus suggesting a potential time lag needed to observe the consequences of gene expression even at protein level.

As for gene expression, the two different primer pairs, previously used by Soglia et al. (2020), successfully amplified the *VIM* common sequence and the *VIM* long-isoform, thus validating that both *VIM* sequences are commonly expressed not only in FG but also in MG chickens. As previously reported, the *VIM* long-isoform differs from the common sequence by having a longer promoter and exon one regions (Soglia et al., 2020). Interestingly, in humans these regions of *VIM* gene were recently demonstrated to bind proteins playing important roles in the transcription regulation of other genes and proteins implicated in protein synthesis (e.g., the eukaryotic elongation factor-1 complex; eEF-1), cell migration and extracellular matrix remodeling (Al-Maghrebi et al., 2002; Pisani et al., 2016; Ostrowska-Podhorodecka et al., 2022). Therefore, dissimilarities between the two *VIM* sequences at the promoter level might suggest differences in regulating protein synthesis. In addition, it was also demonstrated that *VIM* exerts a role in contrasting cellular stress conditions (such as those induced by the accumulation of misfolded proteins) by interplaying with misfolded aggregates and interacting with proteins involved in the inflammatory response (Pattabiraman et al., 2020). Given the above and considering the numerous signs of stressful conditions (e.g., oxidative stress, inflammatory stress, and endoplasmic reticulum stress) observed in FG broilers, especially those affected by growth-related abnormalities (Abasht et al., 2016; Papah et al., 2018; Pampouille et al., 2019), a possible involvement of VIM in contrasting cellular stress may be hypothesized. According to this hypothesis, the remarkable increase in VIM heterodimeric form observed at d 21 might be attributed to muscle regeneration from one side and it might also represent a compensatory mechanism aimed at contrasting cellular stress in FG muscles. Moreover, the differences in *VIM com* observed in FG during the growth period and between FG and MG broilers at d 28, d 35 and d 42 suggest that this isoform may belong to one or more transcripts having a role not only in muscle regeneration but also in contrasting cellular stressful events. The role of VIM protein in protecting mitochondria during oxidative stress has been demonstrated in cultured cells, and mutations causing aminoacidic substitution in specific VIM sites or isoforms lacking some specific sequences in the N-terminal were found to cause a loss of VIM protective ability on mitochondria (Matveeva et al., 2010). In agreement with that, different variants of *VIM* transcripts were demonstrated to have diverse functions (Danielsson et al., 2018). Considering the opposite trend of the two *VIM* sequences as the growth proceeded, different roles between *VIM com* and *VIM long* during the animal growth could be hypothesized. In detail, a potential involvement of *VIM com* in the development of growth-related abnormalities in FG might be hypothesized whereas *VIM long* might not have substantial roles in the mechanisms underlying broilers’ abnormalities.

As mentioned above, the *VIM* gene may regulate extracellular matrix remodeling through post-transcriptional regulatory pathways concerning collagen synthesis (Ostrowska-Podhorodecka et al., 2022) and fibroblast proliferation (Danielsson et al., 2018). Differences in the expression level of *VIM* between FG and MG birds have been detected since the early stages of growth. In this respect, considering the important role of VIM in SCs proliferation (Vater et al., 1994), the high level of *VIM com* observed in FG at d 7 might be partially related to the growth rates of these genotypes and to SCs’ activity, which reaches its maximum level during the first week post-hatch (Halevy et al., 2000; Daughtry et al., 2017; Velleman and Song, 2017; Velleman et al., 2019). Moreover, since *VIM* was found to play a key role in protecting stem cells during proliferation (Pattabiraman et al., 2020), its potential involvement in counteracting the development of cellular stress during SCs proliferation and muscle regeneration could be hypothesized. To support this hypothesis, it is worth mentioning that selection for increased growth rate and muscle mass accretion resulted in a hypertrophic growth of the fibers and concurrently favored the development of thicker PM, which are however perfused by an insufficient circulatory supply (Sihvo et al., 2018; Pampouille et al., 2019). Under these circumstances, the SCs function and muscle repair mechanism may be hindered (Velleman, 2019) thus contributing to explain the severe histological lesions observed in FG broilers from d 35 to d 42. Overall, these findings further corroborate the hypothesis of a strong association between SCs proliferation (which is necessarily required for muscle fiber regeneration) and the expression and subsequent synthesis of VIM (Vater et al., 1994). In addition, VIM was found to exert a profound effect on fibroblasts’ proliferation thus leading to collagen production from one side and TGF-β secretion from the other (Velleman, 2019). The expression of this last transforming growth factor may be further exacerbated under hypoxic conditions, frequently occurring in FG (Abasht et al., 2016; Malila et al., 2019; Pampouille et al., 2019; Lake and Abasht, 2020) and, in its turn, regulates decorin expression during muscle growth, which is essential for collagen fibril formation and crosslinking. In addition, along with decorin, TGF-β is involved in the modulation of muscle fibrosis by means of regulating connective tissue growth factors (Velleman et al., 2019). Overall, VIM was found to have the ability to tip the balance from the regenerative process to the fibrotic repair (Walker et al., 2018). In this regard, given the above and considering the upregulation of the genes encoding for decorin and TGF-β previously observed in wooden breast-affected muscles (Velleman and Clark, 2015), it seems reasonable to hypothesize a regulatory role of VIM in this whole process, with special regard to the *VIM com* sequence at the gene level. On the other hand, the progressive decline in the amount of *VIM long* sequence and VIM protein observed in FG broilers respectively from d 14 and d 21 onward might be explained by considering that VIM synthesis gradually decreases with myotubes maturation (Bornemann and Schmalbruch, 1992). This trend was observed both in FG and MG with the last exhibiting an increase in VIM protein at d 35 (even exceeding that assessed in FG), likely ascribable to their growth-rate which may have slowed down the onset of muscle degeneration and subsequent regeneration. The regeneration processes likely ceased or being impaired, became largely ineffective during the developmental stages corresponding to the achievement of market weight (i.e., d 35 and d 42) so that no further variations in VIM content were observed in FG broilers. Accordingly, the progressive decline of the *VIM* long-isoform seemed to be associated with the physiologic role of this protein aimed at maintaining cell architecture, adhesion and migration (Danielsson et al., 2018). Overall, results obtained for VIM protein agreed with those of the *VIM long* gene in which a progressive decline of its normalized quantification was observed in FG and no differences were found between the two genotypes at d 35 and d 42. On the contrary, considering the significant differences in the normalized expression level of *VIM com* observed between FG and MG at d 35, along with the tendency toward significance at d 28 and d 42, the common sequence of the *VIM* gene may be considered a reliable marker of the regenerative processes in FG broilers, at least from d 35 onwards. On the whole, the present results seemed to support the hypothesis that different *VIM* transcripts may determine the synthesis of proteins having diverse biological roles, as previously described in humans (Zhou et al., 2010), and that *VIM long* may not be directly related to muscle regeneration, as opposed to what hypothesized for *VIM com*.

The outcomes of the investigations carried out to quantify native DES and its heterodimeric form exhibited analogous and fluctuating trends in which an increased synthesis of this protein is then followed by a sharp decline in its content in the subsequent sampling age. These findings perfectly matched with the events related to VIM thus confirming their sequential and differential synthesis during the regeneration processes taking place within the PM. Indeed, once synthesized, DES integrates into the pre-existing VIM filaments and, as a consequence of their progressive decline, ultimately results in the development of a DES-dominated network (Cary and Klymkowsky, 1994). Accordingly, a co-expression of VIM and DES was observed by means of IHC both within the connective tissue composing the endomysial and perimysial spaces and in the fibers. Indeed, some fibers were found to be immunoreactive to both VIM and DES (thus demonstrating their co-expression) whereas others exhibited a distinct reaction for one protein rather than the other.

Although there is still an ongoing debate, since its biochemical identification at the end of 1970s, different functions have been ascribed to DES. Among the others, DES was demonstrated to exert a primary role in maintaining sarcomeres’ alignment (Morgan, 1990) and, in light of being one of the first muscle-specific genes expressed during development, it was also hypothesized to play a biological role in muscle development (Rudnicki et al., 1993). Indeed, desmin-null mice showed impaired myoblast fusion and myotube formation (Hnia et al., 2015) along with altered nuclear and mitochondrial shape/positioning (Paulin and Li, 2004; Capetanaki et al., 2007). Not surprisingly, DES was also found to regulate a broad spectrum of cellular processes by serving as a platform for the integration of signals between the outside and the inside of some organelles, such as the nucleus and mitochondria (Clemen et al., 2013; Hnia et al., 2015). Given the above, the distribution and expression of DES were investigated in muscular disorders (e.g., Duchenne Muscular Dystrophy) affecting humans and other species (Gallanti et al., 1992; Fröhlich et al., 2016), and it was considered a reliable marker of muscle regeneration under pathological conditions. Thus, the fluctuating trends observed for both native DES and its heterodimeric form might be due to the degenerative and subsequent regenerative processes occurring in PM of FG chickens. Muscle degeneration, implying fibers’ necrosis and loss of myofibrils, likely resulted in DES degradation followed by an increased synthesis of this protein to support the structural organization of the regenerating fibers. In this regard, it is worth mentioning the lack of correspondence between *DES* gene and its encoded protein at d 42 when a 2-fold higher expression of *DES* gene was found in FG compared with MG, whilst no differences were found at the protein level. The higher transcriptional level of *DES* observed in FG at d 42 could be likely due to an up-regulation aimed at synthesizing the degraded DES protein and thus re-establishing the structural organization of the regenerating fibers. In addition, the discrepancy in the protein content may be imputable to a potential time lag needed for the translation of the DES protein thus determining a delay in the observation of the consequences of the gene expression at the protein level. In addition, although not significant, the increasing trend observed for *DES* gene in FG over the growth period was consistent with the differential synthesis of this protein, which progressively integrates and replaces the pre-existing VIM filaments (Cary and Klymkowsky, 1994). Indeed, the opposite general trends of *VIM long* and *DES* observed in FG from d 7 to d 42 perfectly overlapped with the evidence that *VIM* expression is downregulated as muscle regeneration proceeds and/or during myotubes’ maturation whereas *DES* level increases (Hnia et al., 2015).

Regarding the role of DES in muscle regeneration processes, Soglia et al. (2020) evidenced an increased abundance both at the gene and protein level of DES in chickens affected by growth-related abnormalities, thus proposing its level of expression as a marker for the regenerative phenomena taking place in PM muscles belonging to FG broilers. In this regard, the higher level of *DES* in FG compared with their MG counterpart observed in the present research corroborated the statement that this gene can be effectively exploited as a marker of muscle fibers regeneration in broiler chickens, especially from d 28 onwards. In addition, it is worth mentioning the potential role of DES in regulating mitochondrial morphology and bioenergetic capacity (Kuznetsov et al., 2020) along with the evidence that its defective anchorage and/or spacing profoundly affect these features (Knowles et al., 2002). The abovementioned traits mirrored those observed at ultrastructural level by Sihvo et al. (2018) in the early phase of wooden breast (in 22-days-old broilers), when mitochondrial swelling, vacuolation, and loss of cristae were apparent and associated with endoplasmic reticulum stress (Papah et al., 2018; Sihvo et al., 2018). Therefore, considering the presence of DES aggregates observed within the fibers through IHC, a synthesis and subsequent accumulation of an altered DES protein (Delort et al., 2019) might be hypothesized to occur also in FG broilers.

Our hypothesis concerning the essential role of VIM and DES in the regeneration processes occurring in FG chickens might be further supported by the findings of the correlations existing at gene level. Indeed, the positive correlation between *VIM com* and *DES* found in FG at d 42 may corroborate the hypothesis that *VIM com* could play a role in counteracting stressful conditions (probably exacerbated by an altered DES deposition) occurring in PM. These results agreed with those reported in our previous study performed on broiler chickens (Soglia et al., 2020). Besides, Vater et al. (1994) demonstrated that *VIM* may up-regulate the expression of *DES* at later stages of muscle regeneration, thus contributing to explain their positive correlation at the latest stages of PM development in broiler chickens prior to slaughter (i.e., d 42). Noteworthy, at d 35, *DES* was positively correlated with the *VIM* long-isoform: since the normalized expression of *VIM long* decreased during the growth period, a tendency to a decreased expression of *DES* might be hypothesized, which is then mirrored by the decreased protein amount identified at d 42.

In conclusion, the present study represented the first attempt to investigate the expression and distribution of VIM and DES over the growth period (from 7 to 42 days of age) in FG and MG broiler chickens and confirmed their potential use as markers of the regenerative processes occurring in skeletal muscle. In detail, the findings of the present research seem to support the existence of a relationship between the occurrence of muscle regeneration and the growth rate of the meat-type chickens with the FG hybrids being more susceptible to this phenomenon (as well as to the occurrence of growth-related muscle defects). This evidence may be of a relevant importance when considering that VIM and DES could be potentially exploited as molecular markers to identify breeders bearing/prone to develop the growth-related abnormalities and exclude them from the breeding practices, thus improving the economic and environmental sustainability of the system. In addition, this study allowed to ascertain the key function of VIM in coordinating the sequence of events occurring during muscle regeneration and shed light on the biological roles of DES in preserving the stability of the sarcomeric structure as well as its involvement in several cellular processes. In this context, considering the similarities existing between broilers’ abnormalities and other disorders affecting humans (such as desminopathies as well as vimentin-related myopathies) *Gallus* may be potentially proposed as a spontaneous alternative animal model for studying the pathogenesis of these conditions which currently requires the use of artificially induced laboratory animals.

## Data Availability

The raw data supporting the conclusions of this article will be made available by the authors, without undue reservation.
