# Supplementary material for: The evolution of vimentin and desmin in Pectoralis major muscles of broiler chickens supports their essential role in muscle regeneration
Source: Front Physiol. 2022 Sep 5;13:970034. doi: 10.3389/fphys.2022.970034 (PMC9483144; doi:10.3389/fphys.2022.970034)
Supplement: Supplementary file 1 [file Table1.DOCX]

Supplementary Material

**Supplementary Table 1.** Summary of the primers used for the gene expression quantitation.

| **Gene name** | **Acronym** | **Primers** | **Amplicon length (bp)** |
| --- | --- | --- | --- |
| *Vimentin (long transcript)* | *VIM long* | F: 5’- TGCCTATTCCAACCTTTGCT -3’  R: 5’- ATCGTGATGCTGGGAAGTTT-3’ | 152 |
| *Vimentin (common sequence)* | *VIM com* | F: 5’- CGCTACATCACGTCCAGCAC -3’  R: 5’-GTCCGCCAGGGTGAAGTC -3’ | 177 |
| *Desmin* | *DES* | F: 5’-GCGTGACAACCTGCTAGATG-3’  R: 5’-GCTCTGAAAGCAGCCAAGTT-3’ | 102 |
| *Glyceraldehyde-3-phosphate dehydrogenase* | *GAPDH* | F: 5’- TGACAGCCATTCCTCCAC-3’  R: 5’- TGGACCATCAAGTCCACAAC -3’ | 126 |
| *Ribosomal protein L4* | *RPL4* | F: 5’ -CTGAACCCATACGCCAAAAC- 3’  R: 5’ – TGGCTTTCTTCTCCTCCTTG-3’ | 85 |
| *Ribosomal protein lateral stalk subunit P0* | *RPLP0* | F: 5’- CCATCGTCAATGGCTACAAG-3’  R: 5’- GAACGCCTTCACCTTTTCAG-3’ | 86 |
