# Supplementary material for: The evolution of vimentin and desmin in Pectoralis major muscles of broiler chickens supports their essential role in muscle regeneration
Source: Front Physiol. 2022 Sep 5;13:970034. doi: 10.3389/fphys.2022.970034 (PMC9483144; doi:10.3389/fphys.2022.970034)
Supplement: Supplementary file 2 [file Table2.DOCX]

Supplementary Material

**Supplementary Table 2**. Summary of the amplification conditions of target and normalizing genes using the *Real-Time* quantitative PCR (RT-qPCR) analysis.

| **Gene name** | **Acronym** | **RT-qPCR conditions** | | **Melting Temperature °C** |
| --- | --- | --- | --- | --- |
|  |  | **Denaturation** | **Annealing/Extension** |  |
| *Vimentin (long transcript)* | *VIM long* | 95°C; 15 sec | 60°C; 20 sec | 80-87°C |
| *Vimentin (common sequence)* | *VIM com* | 95°C; 15 sec | 60°C; 20 sec | 80-87°C |
| *Desmin* | *DES* | 95°C; 15 sec | 60°C; 20 sec | 80-87°C |
| *Glyceraldehyde-3-phosphate dehydrogenase* | *GAPDH* | 95°C; 10 sec | 63°C; 20 sec | 80-87°C |
| *Ribosomal protein L4* | *RPL4* | 95°C; 15 sec | 57°C; 20 sec | 77-87°C |
| *Ribosomal protein lateral stalk subunit P0* | *RPLP0* | 95°C; 15 sec | 61°C; 20 sec | 77-87°C |
