# Supplementary material for: The evolution of vimentin and desmin in Pectoralis major muscles of broiler chickens supports their essential role in muscle regeneration
Source: Front Physiol. 2022 Sep 5;13:970034. doi: 10.3389/fphys.2022.970034 (PMC9483144; doi:10.3389/fphys.2022.970034)

Supplementary Figure S3. Representative immunoblots obtained for vimentin and desmin clearly exhibiting the presence of two specific bands.

Vimentin

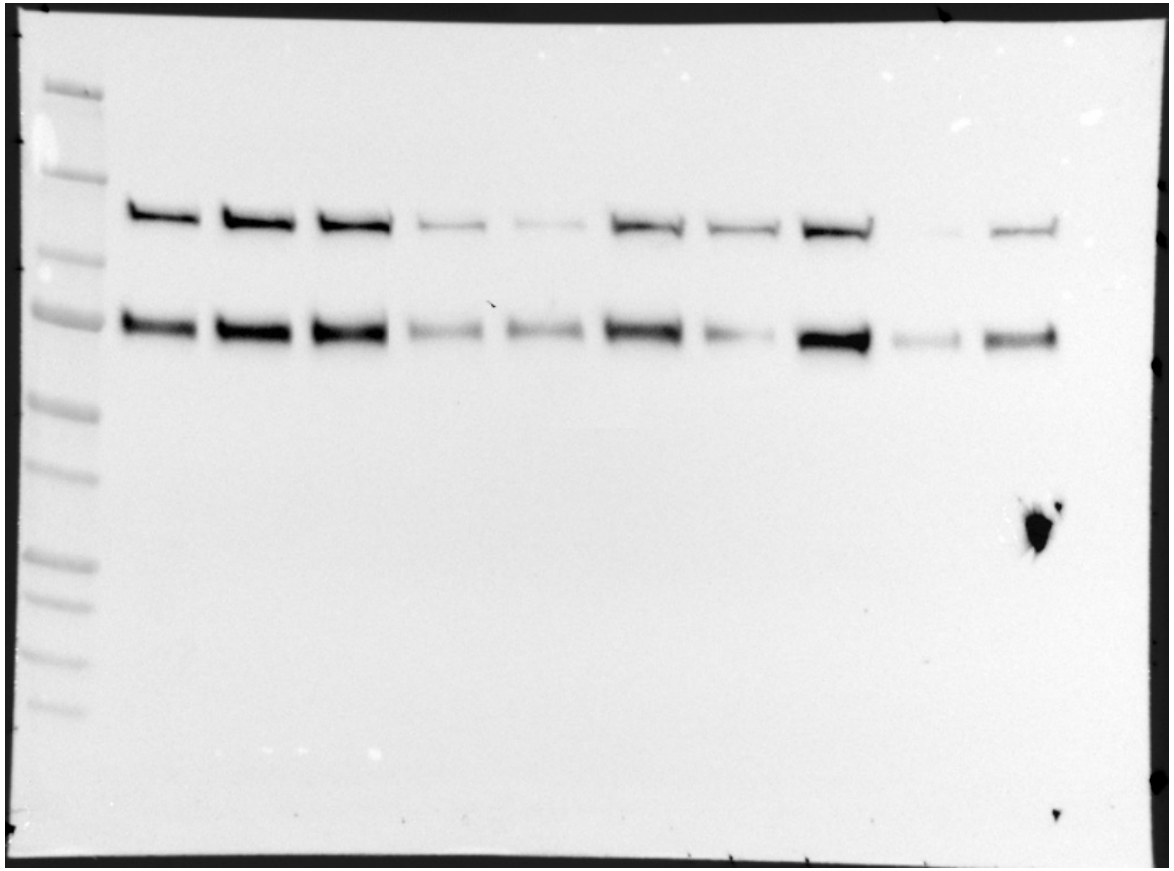

Desmin

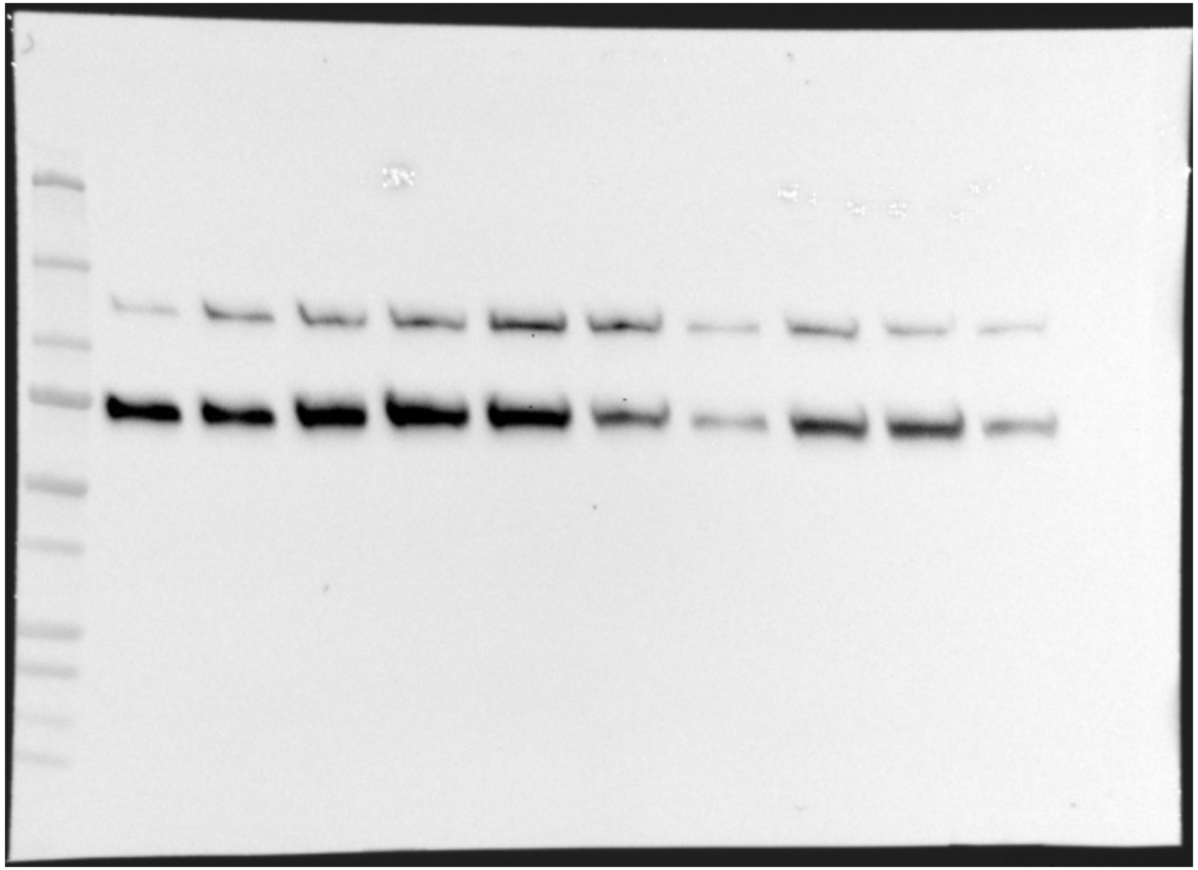

Supplement: Supplementary file 3 [file DataSheet1.PDF]
